# Effects of intensive insulin therapy combined with low molecular weight heparin anticoagulant therapy on severe pancreatitis

**DOI:** 10.3892/etm.2014.1694

**Published:** 2014-04-25

**Authors:** JUN-DONG DU, XI ZHENG, ZHI-QIANG HUANG, SHOU-WANG CAI, JING-WANG TAN, ZHAN-LIANG LI, YONG-MING YAO, HUA-BO JIAO, HUI-NAN YIN, ZI-MAN ZHU

**Affiliations:** 1 Departments of Heptapobiliary Surgery, The First Affiliated Hospital to General Hospital of PLA, Beijing 100048, P.R. China; 2 Division Three for Senior Officers, The First Affiliated Hospital to General Hospital of PLA, Beijing 100048, P.R. China; 3 Department of Hepatobiliary Surgery, The General Hospital of PLA, Beijing 100853, P.R. China

**Keywords:** intensive insulin therapy, low molecular weight heparin anticoagulant therapy, severe acute pancreatitis, albumin, white blood cell, diastase, intestinal function recovery time

## Abstract

The current study explored the effects of intensive insulin therapy (IIT) combined with low molecular weight heparin (LMWH) anticoagulant therapy on severe acute pancreatitis (SAP). A total of 134 patients with SAP that received treatment between June 2008 and June 2012 were divided randomly into groups A (control; n=33), B (IIT; n=33), C (LMWH; n=34) and D (IIT + LMWH; n=34). Group A were treated routinely. Group B received continuous pumped insulin, as well as the routine treatment, to maintain the blood sugar level between 4.4 and 6.1 mmol/l. Group C received a subcutaneous injection of LMWH every 12 h in addition to the routine treatment. Group D received IIT + LMWH and the routine treatment. The white blood cell count, hemodiastase, serum albumin, arterial partial pressure of oxygen and prothrombin time were recorded prior to treatment and 1, 3, 5, 7 and 14 days after the initiation of treatment. The intestinal function recovery time, incidence rate of multiple organ failure (MOF), length of hospitalization and fatality rates were observed. IIT + LMWH noticeably increased the white blood cell count, hemodiastase level, serum albumin level and the arterial partial pressure of oxygen in the patients with SAP (P<0.05). It markedly shortened the intestinal recovery time and the length of stay and reduced the incidence rate of MOF, the surgery rate and the fatality rate (P<0.05). It did not aggravate the hemorrhagic tendency of SAP (P>0.05). IIT + LMWH had a noticeably improved clinical curative effect on SAP compared with that of the other treatments.

## Introduction

Severe acute pancreatitis (SAP) is a common cause of acute abdominal pain that is attributed to pancreatic activation caused by numerous factors. SAP is primarily manifested by local pancreatic hemorrhage and necrosis. It is accompanied by multiple organ failure (MOF) and has a high fatality rate (1). With regard to the origin of SAP, the theory of microcirculation dysfunction (MCD) is widely accepted since MCD is involved in the development of SAP; the classic theory of pancreatic autodigestion is an alternative (2). MCD, caused by pancreas-nourishing vessel thrombosis, is one of the primary causes of pancreatic ischemia and necrosis (3). Although low molecular weight heparin (LMWH) anticoagulant therapy improves the prognosis of SAP (4), the fatality rate due to infections and MOF during treatment remains as high as 15–40% (5). A large-scale clinical observation study (1,548 cases) demonstrated that intensive insulin therapy (IIT) greatly reduces the incidence of MOF and associated fatality rates in SAP patients in intensive care units (6). However, to the best of our knowledge, the curative effect of IIT combined with LMWH on SAP has not yet been reported.

In 2008, the Department of Heptapobiliary Surgery, the First Affiliated Hospital to General Hospital of PLA (Beijing, China), adopted IIT + LMWH anticoagulation for patients with SAP and achieved satisfactory results. This study focused on an investigation into improving the effects of this combined method on SAP and aimed to find a more effective treatment method for SAP.

## Materials and methods

### Patients

The inclusion criteria included: i) diagnosis based on the revised scheme of the North American Clinical Diagnostic and Staging Standards of Acute Pancreatitis in 2008 (7); ii) inpatients aged from 18 to 85 years; iii) no history of diabetes; and iv) no participation in other clinical studies within the previous 3 months. Patients not meeting these inclusion criteria or those with a severe medical disease or cancer cachexia were excluded from this study. The study was conducted in accordance with the Declaration of Helsinki and with approval from the Ethics Committee of the First Affiliated Hospital to General Hospital of PLA. Written informed consent was obtained from all participants.

A total of 134 patients with SAP receiving treatment at the First Affiliated Hospital to General Hospital of PLA between June 2008 and June 2012 were enrolled in this study. They were divided randomly into groups A (control; n=33), B (IIT; n=33), C (LMWH; n=34) and D (IIT + LMWH; n=34), according to a random number table generated from their admission sequence. No significant differences regarding age, gender, acute physiology, chronic health evaluation (APACHE II) scores on admission or disease constitution were observed among the groups (P>0.05; Table I).

### Treatment methods

Group A (n=33) received routine treatment for SAP, including intravenous nutritional support, fasting and water deprivation, continuous gastrointestinal decompression, spasmolysis and analgesia, acid suppression, pancreatic secretion inhibition with octreotide and the intravenous administration of broad-spectrum antibiotics. All patients were administered ectogenic insulin in a 1:6–8 glucose:insulin ratio for blood glucose control. The required insulin was infused with liquid and a subcutaneous injection was given when necessary to maintain the blood glucose level at between 6.0 and 11.1 mmol/l.

Group B (n=33) received continuous intravenous pumped insulin (50 U with 50 ml physiological saline) as well as the routine treatment. Blood glucose was monitored (initially once per h) and the insulin pumping speed was adjusted to maintain the glucose level at between 4.4 and 6.1 mmol/l.

Group C (n=34) received a subcutaneous injection of 5,000 U LMWH once every 12 h once no contradictions of anticoagulant therapy were confirmed. LMWH was administered in addition to the routine treatment.

Group D (n=34) received continuous intravenously pumped insulin (50 U with 50 ml physiological saline) in addition to the routine treatment. Blood glucose was monitored (initially once per h) and the insulin pumping speed was adjusted to maintain the glucose level at between 4.0–6.1 mmol/l. In addition, 5,000 U LMWH was subcutaneously administered once every 12 h.

### Curative effect evaluation

A number of examinations were performed prior to treatment and after 1, 3, 5, 7 and 14 days of treatment. These were a routine blood examination to determine the white blood cell count, a venous blood biochemical examination to determine the levels of hemodiastase and serum albumin, a venous blood chemical examination to determine the prothrombin time and blood gas analyses using radial artery puncture to obtain arterial partial pressures of oxygen. The results of these assays were then compared.

The intestinal function recovery time from first admission was recorded, taking abdominal distension disappearance, normal bowel sounds, self flatus passing and defecation and diet restoration as the signs of normal function recovery. The length of stay was the time period from the initiation of systemic treatment to full recovery, discharge from the hospital or mortality.

MOF was diagnosed based on the diagnostic criteria of MOF by Goris (8) and incidence rates were calculated. Patients in whom the pathogenic condition progressed or for whom surgery was necessary (due to the occurrence of abdominal infection or hemorrhage) were recorded for calculation of the surgery rate.

Fatality rates were calculated according to the mortalities caused by complications due to SAP, including shock, MOF and abdominal infection.

### Statistical analysis

Data are presented as mean ± standard deviation and were analyzed using SPSS software, version 16.0 (SPSS, Inc., Chicago, IL, USA). ANOVA was used to compare homogenous data for variance among groups and rank tests for any heterogeneous data. Factor analysis was performed to determine the synergetic effect between IIT and LMWH. P<0.05 was considered to indicate a statistically significant result.

## Results

### White blood cell counts

Prior to treatment and at day 1 of treatment, no significant differences in the white blood cell count were observed among the groups. At day 3, the white blood cell counts in groups C and D were significantly lower compared with those in group A (P<0.05). At days 5, 7 and 14, the counts of groups B and C exhibited significant differences compared with those of groups A and D (P<0.05), whereas no significant differences were observed between groups B and C (P>0.05). The results are summarized in Table II.

### Hemodiastase levels

Prior to treatment and at day 1 of treatment, no significant differences in the hemodiastase levels were observed among the groups. At day 3, the hemodiastase levels of groups C and D were significantly lower compared with those in group A (P<0.05). At days 5, 7 and 14, the hemodiastase levels of groups B and C exhibited significant differences compared with those of groups A and D (P<0.05), whereas no significant differences were observed between groups B and C (P>0.05). The results are summarized in Table III.

### Serum albumin levels

Prior to treatment and at days 1 and 3 of treatment, no significant differences in the serum albumin levels were observed among the groups. At days 5, 7 and 14, the albumin levels of groups B, C and D showed significant differences compared with those of group A (P<0.05). Although the albumin levels of groups B and C did not exhibit a significant difference when compared with one another (P>0.05), the albumin levels of group D were significantly higher compared with those of groups B and C (P<0.05). The results are summarized in Table IV.

### Prothrombin time

Prior to treatment and at days 1 and 3 of treatment, no significant differences in prothrombin times were observed among the groups (P=0.0518). At days 5, 7 and 14, the prothrombin times of groups A and C did not show a significant difference. In comparison, groups B and D exhibited significant differences (P<0.05). The results are summarized in Table V.

### Arterial partial pressures of oxygen

Prior to treatment and at day 1 of treatment, no significant differences in the arterial partial pressures of oxygen were observed among the groups. At days 3, 5, 7 and 14, groups B, C and D showed significantly higher arterial partial pressures of oxygen compared with those in group A (P<0.05). However, groups B and C did not show significant differences. At days 5 and 7, the arterial oxygen partial pressures in group D were observed to be significantly higher compared with those in groups B and C (P<0.05). The results are summarized in Table VI.

### Other indices

Groups B, C and D showed significant differences regarding the mean length of stay, the intestinal function recovery time, the incidence rate of MOF, the surgery rate and the fatality rate compared with group A (P<0.05). Group D exhibited significant differences compared with groups B and C (P<0.05) whereas no significant differences were observed between groups B and C (P>0.05). The results are summarized in Table VII.

## Discussion

SAP is primarily attributed to abnormal pancreatic activation caused by various factors. The activated pancreatic tissue releases cytokines into the blood, which activates the cytokine receptors of the pancreas and remote organs. Such cascade reactions of the cytokines may be responsible for the development of pancreas-limited inflammatory reaction into systemic inflammatory reaction and MOF (9). With regard to the origins of SAP, in addition to the classic pancreatic autodigestion theory, the theory of MCD has also been widely accepted by scholars (10). The pancreas is sensitive to ischemic injury (2) as the arteriospasm contained in the pancreatic lobules manifests spasms and microthrombosis under the actions of bile reflux, oxygen radical stimulation and endogenous nitric oxide reduction in SAP. This further leads to ischemic injury of the lobules. In addition, SAP is often accompanied by abnormalities in the third space, body fluid loss in great quantities and an increase in the blood viscosity. This, together with the release of a large amount of inflammatory factors and the activation of the blood coagulation system, maintains the blood in a hypercoagulable state. The state is likely to induce pancreatic microthrombosis and cause necrosis of the pancreatic tissues, thereby exacerbating systemic inflammatory reactions. As such, the correction of pancreatic ischemia and the improvement of pancreatic microcirculation at an early stage have become important therapeutic measures for SAP (11).

Apart from systemic inflammatory reactions, patients with SAP also tend to present with hypermetabolic reactions primarily characterized by stress hyperglycemia. This is primarily attributable to the increase in catabolism-promoting hormones, such as glucocorticoids, glucagon, growth hormones, catecholamine (12), cytokines and other inflammatory mediators, which cause reductions in the glucose uptake and utilization capacities triggered by peripheral insulin, namely insulin resistance (IR). Hyperglycemia following IR has a powerful inflammation-promoting effect on the organism in a stressed state. Continuous hyperglycemia results in increased oxidative stress for mononuclear macrophages, causing them to release inflammatory cytokines (13). Consequently, the number of inflammatory mediators increases (14) and the pathogenic condition of SAP is aggravated. Therefore, blood glucose control is of a particular significance for improving the prognosis of SAP.

LMWH inhibits platelet aggregation and reduces the probability of the transformation of temporary platelet clots into permanent clots. This is achieved by inhibiting thrombin and the coagulation active factor Xa in order to change blood rheological characteristics, reduce blood viscosity and improve microcirculation (15). The administration of a large dose of insulin, by way of IR-caused stress hyperglycemia control after serious injury, reverses the negative nitrogen balance, promotes tissue healing and reduces the incidence rate of infections and the fatality rate (16). For these reasons, IIT has become a hotspot for research in recent years. Although IIT has an inhibitory effect on the inflammatory mediators of SAP (17), to the best of our knowledge, the curative effect of IIT + LMWH anticoagulant therapy has not been reported.

In this study, SAP patients were divided into different groups and received IIT, LMWH anticoagulant treatment or IIT + LMWH, respectively. The curative effects were then compared. The results revealed that LMWH anticoagulant treatment reduced the white blood cell count and hemodiastase level and increased the blood oxygen saturation at the early stage (after days 1 and 3 of treatment). Although the LMWH anticoagulant treatment group did not show significant differences from the IIT group at the late stage, the LMWH treatment exhibited improved curative effects when compared with the routine treatment. In comparison with the single treatment group, the combined group exhibited markedly reduced values of white blood cell count and hemodiastase level. This finding was consistent with microcirculation dysfunction. The underlying reason may be as follows. At the early stage of SAP, LMWH improves microcirculation and reduces the adhesion and migration of white blood cells in tissues through its direct anticoagulant effect. This further reduces the oxidative damage and cytokine production caused by white blood cells, thereby exerting a protective effect. During disease progression, metabolic disorders primarily represented by hyperglycemia occur. IIT maintains the blood glucose level within a normal range to reduce the inflammation-promoting effect of hyperglycemia and promote the restoration of pancreas cells. Furthermore, insulin in itself has anti-inflammatory effects *in vivo*, including an anti-TNFα effect (18) and anti-macrophage retardation factor effect (19), which decrease the levels of white blood cells and hemodiastase. Insulin, as the most important synthesis-promoting hormone *in vivo*, corrects high catabolism and negative nitrogen balance, reduces nitrogen loss, promotes protein synthesis and improves glucose oxidation utilization (20).

Serum albumin levels may be used an index for metabolic evaluation (21). This is demonstrated in the present study as the serum albumin level increased in the combined treatment group. The pancreatic pathological changes and stress state in SAP patients may result in systemic inflammatory response syndrome, which may further lead to multiple organ dysfunction, or even MOF causing mortality (22). In the present study, anticoagulant therapy for microcirculation improvement and IIT for blood glucose control were utilized in combination. This combined therapy improves systemic functions by inhibiting the production of inflammatory factors and activating anti-inflammatory factors (23). This point was demonstrated by the results of the present study. At the early stage of SAP, a great quantity of cytokine-containing exudates, together with histonoxia and local inflammatory reactions, cause gastrointestinal edema and feeble peristalsis of the smooth muscle. In addition, SAP causes cytotoxicity infiltration into retroperitoneal nerve plexuses, leading to intestinal nervous reflex disorders. The joint action of these two factors induces intestinal motor dysfunction and ultimately leads to enteroplegia. The intestinal mucous membrane barrier is destroyed and flora translocation takes place. Consequently, peritoneal infection, fluid and electrolyte imbalance and acid-base imbalance occurs (24). The present study also showed that the combined therapy greatly shortened the intestinal function recovery time and the length of stay. The underlying reason may be that IIT, combined with anticoagulant therapy, inhibits inflammatory reactions by improving metabolism. In doing so, it effectively improves gastrointestinal blood circulation, reduces the damage to the gastrointestinal mucous membrane barrier, promotes the excretion of intestinal endotoxins, reduces bacterial and toxic translocation and alleviates enterogenic infection caused by bacterial translocation and endotoxic uptake (25). These effects may further mitigate intestinal edema and promote the restoration of intestinal peristalsis.

In addition, the current study revealed that the combined treatment group had a markedly decreased fatality rate. A previous study, conducted by the authors of the present study, has demonstrated that IIT improves the prognosis of patients with injury and with pyemia and decreases their fatality rates (26). Another study has reported that LMWH noticeably improves the prognosis of hyperlipidemic pancreatitis and that the underlying mechanisms may be correlated with the improvement of microcirculation, catabolism and anabolism, and the promotion of anti-inflammatory reactions (27). The results of the present study were in line with these findings.

Based on the working mechanisms of IIT and LMWH anticoagulant therapy, a synergetic effect between them is predictable, but with somewhat different action time points. Therefore, IIT combined with LMWH for SAP noticeably improves pancreatic microcirculation, effectively prevents systemic inflammatory reactions, promotes the metabolism of glucose and proteins, prevents MOF, shortens healing time, decreases the fatality rate and improves prognosis. However, laboratory and theoretical studies on the mechanisms underlying the synergetic effect remain to be explored.

## Figures and Tables

**Table I:** Clinical characteristics of the treatment and control groups prior to treatment.

Group	Gender		Age	Disease constitution			APACHE II score
	
	Male	Female		Biliary	Alcoholic	Hyperlipidemic	
A	16	17	49.5±9.4	24	6	3	9.15±3.42
B	18	15	51.5±10.2	23	7	3	9.21±3.46
C	17	17	50.7±9.6	25	7	2	9.13±3.25
D	18	16	51.2±9.8	24	7	3	9.42±3.41
P-value	0.9028		0.3841		0. 8360		0.9198

**Table II:** Comparison of the white blood cells counts between the treatment groups and the control group (10^9^/l).

Group	Pre-treatment	Day 1	Day 3	Day 5	Day 7	Day 14
A	18.23±5.05	17.41±4.45	15.21±4.32	13.58±3.24	11.36±2.98	9.03±2.34
B	19.11±5.43	17.54±4.67	13.23±4.05	10.85±2.75	9.39±2.68	7.26±2.25
C	19.45±5.46	17.73±4.75	13.57±4.21	10.87±2.48	9.58±2.72	7.26±2.25
D	18.91±5.03	17.34±4.24	11.23±3.07	8.75±2.21	7.49±2.34	5.47±2.02
P-value	0.3335	0.8693	0.0170	0.0001	0.0001	0.0001

**Table III:** Comparison of the hemodiastase levels between the treatment groups and the control group (μ/l).

Group	Pre-treatment	Day 1	Day 3	Day 5	Day 7	Day 14
A	2349.2±145.5	1967.2±129.3	978.6±45.6	659.4±35.6	449.8±30.8	108.2±14.5
B	2424.5±156.7	1998.4±132.5	667.2±34.5	385.2±28.5	259.9±20.4	98.8±13.7
C	2412.4±142.3	1987.6±127.5	679.3±39.5	388.1±29.4	272.8±21.5	102.7±14.8
D	2425.6±149.8	1986.2±136.4	557.2±31.2	285.2±28.5	152.4±18.6	92.7±12.6
P-value	0.6962	0.2117	0.0001	0.0001	0.0001	0.0001

**Table IV:** Comparison of the serum albumin levels between the treatment groups and the control group (g/l).

Group	Pre-treatment	Day 1	Day 3	Day 5	Day 7	Day 14
A	29.2±3.3	28.4±3.2	30.6±3.6	31.4±3.7	32.8±3.8	34.2±3.5
B	28.5±3.7	28.1±3.5	30.8±3.8	34.2±3.5	34.9±3.4	37.8±3.7
C	29.5±4.2	28.3±3.6	30.1±3.4	33.7±3.4	34.7±3.5	36.9±3.8
D	28.8±3.9	27.9±3.4	31.2±3.7	36.2±3.9	36.9±3.9	38.0±4.1
P-value	0.6870	0.6057	0.7551	0.0001	0.0001	0.0001

**Table V:** Comparison of the prothrombin times between the treatment groups and the control group (sec).

Group	Pre-treatment	Day 1	Day 3	Day 5	Day 7	Day 14
A	19.6±3.3	18.1±3.2	16.6±2.6	15.4±2.7	14.8±1.8	14.2±1.9
B	20.1±3.7	17.8±3.1	15.1±2.4	13.4±2.1	12.9±1.6	11.8±1.7
C	19.7±3.4	18.3±3.2	16.2±2.5	15.2±2.8	14.3±1.2	13.2±1.7
D	19.8±3.6	17.1±3.0	15.6±2.2	13.6±2.6	12.8±1.3	11.2±1.8
P-value	0.4485	0.6121	0.0018	0.0001	0.0001	0.0001

**Table VI:** Comparison of the arterial partial pressures of oxygen between the treatment groups and the control group (mmHg).

Group	Pre-treatment	Day 1	Day 3	Day 5	Day 7	Day 14
A	76.2±4.3	74.4±4.2	78.6±4.6	82.4±4.7	84.8±4.8	91.1±5.1
B	76.5±4.4	75.1±4.5	85.8±4.9	89.2±5.1	92.9±5.4	96.8±5.9
C	75.3±4.6	75.3±4.6	86.1±4.7	89.3±5.0	92.1±5.2	95.9±5.3
D	76.6±4.7	75.8±4.7	86.8±4.9	93.2±4.8	96.9±5.9	96.6±5.6
P-value	0.6905	0.3482	0.0001	0.0001	0.0001	0.0923

**Table VII:** Comparison of the mean length of stay (days), intestinal function recovery time (days), and incidences of MOF, surgery and fatality among the groups.

Group	Length of stay	Intestinal function recovery time	MOF (n)	Surgery (n)	Fatality (n)
A	27.4±3.2	15.5±3.4	7	3	4
B	19.1±2.3^a^	12.5±3.1^a^	3^b^	2^a^	1^b^
C	20.5±2.5^a^	12.2±3.0^a^	3^b^	2^a^	1^b^
D	15.8±2.0^b^	9.5±2.4^b^	1^b^	0^b^	0^b^

a P<0.05,

b P<0.01 compared with the control group (group A).

MOF, multiple organ failure.
